# The Use of Gut Organoids: To Study the Physiology and Disease of the Gut Microbiota

**DOI:** 10.1111/jcmm.70330

**Published:** 2025-02-19

**Authors:** Ya Deng, Xiaolu Yuan, XianMin Lu, Jiangbo Wu, Chen Luo, Ting Zhang, Qi Liu, Siqi Tang, Zhuo Li, Xingyi Mu, Yanxia Hu, Qian Du, Jingyu Xu, Rui Xie

**Affiliations:** ^1^ Department of Endoscopy and Digestive System Guizhou Provincial People's Hospital Guiyang Guizhou China; ^2^ Zunyi Medical University Zunyi Guizhou China; ^3^ The Second Affiliated Hospital of Zunyi Medical University Zunyi Guizhou China; ^4^ Guizhou Medical University Guiyang Guizhou China

**Keywords:** colorectal cancer, inflammatory bowel disease, intestinal disease, intestinal flora, intestinal organoids

## Abstract

The intestinal flora has attracted much attention in recent years. An imbalance in the intestinal flora can cause not only intestinal diseases but also cause a variety of parenteral diseases, such as endocrine diseases, nervous system diseases and cardiovascular diseases. Research on the mechanism of disease is likely to be hampered by sample accessibility, ethical issues, and differences between cellular animal and physiological studies. However, advances in stem cell culture have made it possible to reproduce 3D human tissues in vitro that mimic the cellular, anatomical and functional characteristics of real organs. Recent studies have shown that organoids can be used to simulate the development and disease of the gut and intestinal flora and have a wide range of applications in intestinal flora physiology and disease. Intestinal organoids provide a preeminent in vitro model system for cultivating microbiota that influence GI physiology, as well as for understanding how they encounter intestinal epithelial cells and cause disease. The mechanistic details obtained from such modelling may provide new avenues for the prevention and treatment of many gastrointestinal (GI) disorders. Researchers are now starting to take inspiration from other fields, such as bioengineering, and the rise of interdisciplinary approaches, including organoid chip technology and microfluidics, has greatly accelerated the development of organoids to generate intestinal organoids that are more physiologically relevant and suitable for gut microbiota studies. Here, we describe the development of organoid models of gut biology and the application of organoids to study the pathophysiology of diseases caused by intestinal dysbiosis.

## 1 Introduction

In the past few decades, researchers have conducted many studies on the gut microbiota, which exists in the gut and affects the physiological function of the human gut. On the one hand, commensal bacteria can help maintain intestinal biology through immune responses [1] and promote the renewal of intestinal epithelial cells. On the other hand, gut microbiota disorders can cause a variety of intestinal diseases, such as inflammatory bowel disease [2] and colorectal cancer [3]. However, due to the unique growth environment of the intestinal flora, establishing culture conditions in vitro is difficult. The emergence of organoids undoubtedly provides a new research direction for humanisation. Organ‐like organoids are collections of organ‐specific cell types that develop from stem cells or precursor cells in a manner similar to self‐organisation in vivo, through cell sorting and spatially limited lineage commitment, these organoids possess some of the functions of that organ [4]. From Henry Van Peters Wilson's first attempt in 1907, to the creation of organoids of the intestine, stomach, tongue, salivary glands, liver, pancreas, brain and retina, to the application of organoids in disease [5], salivary gland organoids not only maintain the unique glandular characteristics of mouse and human salivary glands but also replicate gland diversity [6], and cancer organoids such as those of lung cancer and breast cancer have been established for personalised medicine [7]. In particular, investigators have drawn inspiration from the field of bioengineering, a field focused on the design and manufacture of biocompatible materials that can serve as cell‐guided scaffolds. Methods based on innovative biomaterials and advanced engineering have been used to promote tissue regeneration, thereby improving organoid culture techniques to meet human needs. The discovery of organoids opens up the possibility that studying tissue and organ biology, especially in humans, can be challenging because of the accessibility and ethics of samples. For example, scientists recreate the developmental cell trajectory and transcriptional regulation process defined in vivo from human trophoblast stem cells (hTSCs) and related trophoblast organoids [8]. The discovery of brain organoids provides unprecedented opportunities for the study of human brain in vitro [9], and the discovery of cancer organoids allows researchers to study the occurrence and metastasis of tumours in vitro [10]. Therefore, linking organoids with the study of the gut microbiota is necessary.

## 2 The Origin of Intestinal Organoids and Their Simulation of Gut Physiology

### 2.1 Derivation of Intestinal Organoids

**FIGURE 1:**
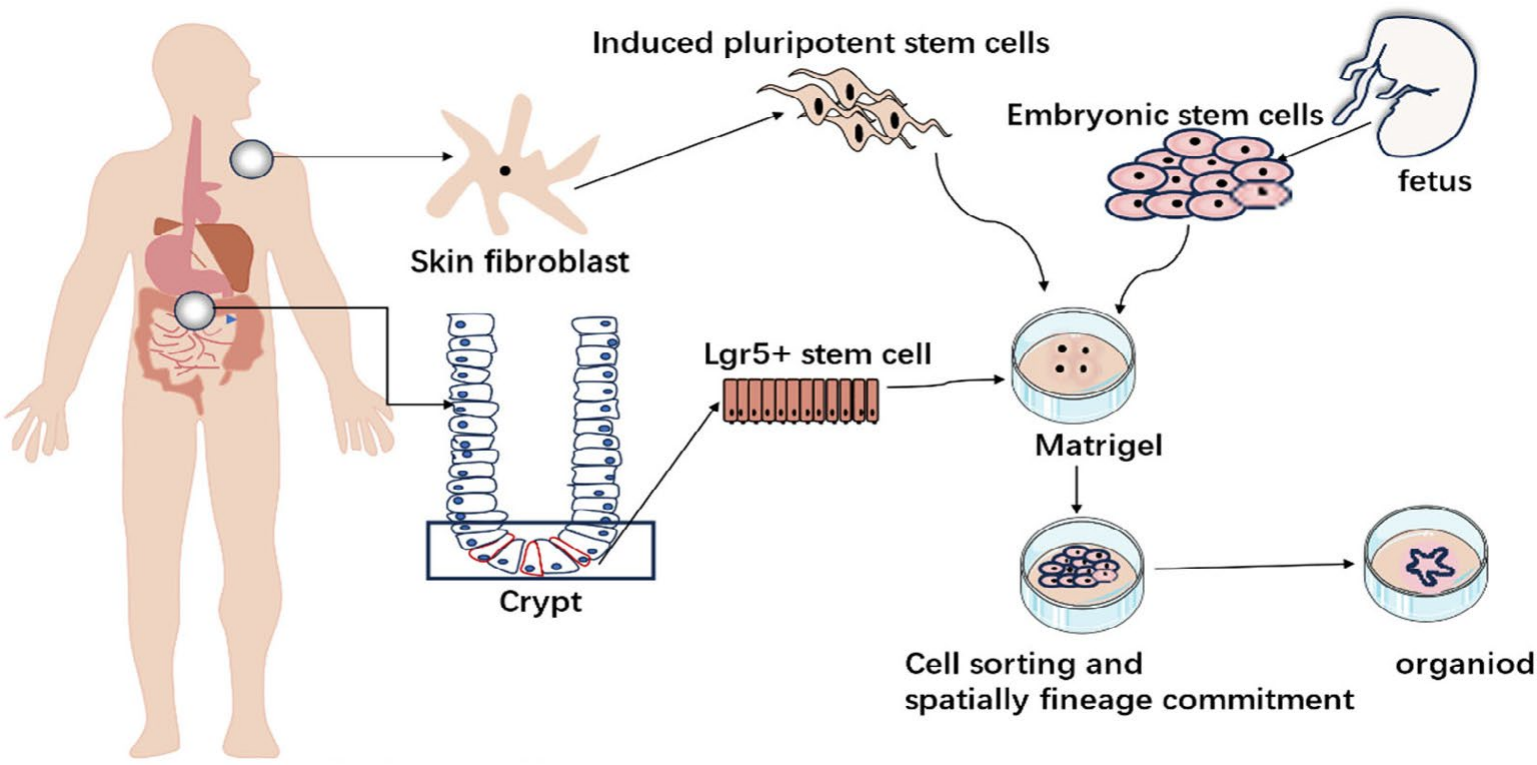
Intestinal organoids. There are two major strategies: Adult stem cell derived organoids, also referred to as, enteroids, Pluripotent stem cell‐ or embryonic stem cell‐derived organoids, also referred to as, human intestinal organoids (HIOs).

3D cell culture technology is a promising approach. There are two types of 3D cell culture models, which can be divided into spheroid and organoid models. Both spheroids and organoids are 3D cellular structures. However, there is a huge difference between spheroids and organoids depending on the cell origin, type and function of the cellular structure implemented in 3D. Non‐stem cell‐based aggregates of primary cells, called spheroids, emerged more than a decade ago, have evolved and become increasingly complex, making it a model for in vitro research [11]. Spheroids are 3D cell culture models with spherical cell units, and many cell types can aggregate to form spheroids [12]. Compared with the existing 2D culture model, it can better simulate physiological conditions and effectively realise and analyse the advantages of cell‐to‐cell interaction. However, because spheroids are sometimes cultured without scaffolds, they exhibit relatively low structural complexity. In addition, spheroids are simple clusters of a variety of cells, such as tumour tissues, embryonic bodies and mammary glands [13]. Cannot self‐assemble or regenerate, so their disadvantage is that they are less biocompatible than organoids. However, tumour spheroids are popular in cancer‐related research because they not only enable 3D cancer cell culture but also enable cell–cell interaction. In particular, tumour spheroids are characterised by mimicking vascularised or poorly vascularised tumours. Multilayer structures can also be formed to realise the outer layer of cells, the middle layer and the inner layer of necrotic cells showing hypoxia. It is also widely used in cancer cell migration and invasion and drug screening research [14]. However, organoids have more complex physiological properties that are closer to in vitro physiology, so we will focus on intestinal organoids next. Intestinal organoids are amongst the most widely studied types of organoids. Intestinal organoids are generated by harvesting single intestinal stem cells (ISCs) expressing lgr5 (Leu‐Repeat‐Containing G‐protein coupled receptor 5), which are rich in crypt cells or isolated from human or mouse small intestine or colon cells. The so‐called adult stem cell‐derived organoids (ASCs) and other organoids are mostly derived from pluripotent stem cells, including 3D intestinal organoids differentiated from induced pluripotent stem cells and embryonic stem cells, iPSCs and ESCs (Figure 1) [15].

### 2.2 Biomaterials for Intestinal Organoid Culture

The media utilised for organoid culture primarily include Matrigel, a natural medium obtained from Engelbreth‐Holm‐Swarm mouse sarcoma cells [16], which is widely considered the “gold standard” scaffold for in vitro cell growth and the first‐generation extracellular matrix (ECM) for organoid culture systems. However, since the properties of medium are mouse‐dependent and vary greatly from one Matrigel to another, the resulting intestinal organoids are unstable and pose a risk of tumorigenesis for organoid implantation. For these reasons, chemically defined hydrogels have recently been cited to support the culture of intestinal organoids by replacing hard‐to‐control natural media. Natural and synthetic hydrogels are potential materials that can be use in Matrigel media. Hydrogels are produced by polymers and can be divided into natural and synthetic hydrogels according to their type. These methods have many advantages. The reported hydrogels, which support the growth and differentiation of intestinal organoids, including natural hydrogels such as alginate, are the minimum hydrogels required to support the growth and differentiation of intestinal organoids. Isamu Ogawa and colleagues developed suspension natural polysaccharide hydrogels that support the growth of intestinal organoids and can be used to measure the activity of drug transporters and drug‐metabolising enzymes in a way that is not available with Matrigel medium [17]. Hyaluronic acid elastomin‐like protein (Help) chitosan and collagen hydrogels are natural hydrogels with potential applications. A recent study identified three key factors of natural hydrogels [18]. First, fibrin is a physical support that provides anchoring molecules such as arg‐gly‐asp (RGD) sequences to adhere to ISCs, which is required for gut stem cell proliferation. Second, the appropriate stiffness of fibrin affects the differentiation of intestinal organoids, with 3–4.5 mg/mL fibrin providing the best stiffness for organoid differentiation. Finally, laminin‐111 is a major biological signal required for growth [19], and some synthetic hydrogels, such as a four‐armed maleimide‐terminated poly(ethylene glycol) macromer [20], recombinant engineered extracellular matrix (eECM) [21], polyethylene glycol, and V‐ORG‐3 [22], are ideal for 3D organoid culture due to their good biocompatibility, independent regulation, repeatability, degradability, and ability to artificially respond to physical stimuli.

## 3 Studies of Intestinal Organoids in Relation to the Gut Microbiota

### 3.1 Aspects of Gut Physiology

Intestinal organoids are a microcosm of normal or diseased epithelium, can be derived from specific tissues, can be established very efficiently from different gastrointestinal regions, have long‐term scalability, and mimic tissue and patient‐specific responses to ex vivo therapies [23]. These finding have been confirmed by the transplantation of small intestinal organoids to the colon surface of immunodeficient mice, in which xenografts of human small intestinal organoids were able to correctly re‐establish the intestinal epithelium and its absorption machinery compared with those of controls, and induced intestinal epithelial maturation, similar to what is seen in the human gut [24]. In addition, combining human intestinal organoids with tissue‐engineered small intestines and transplanting them into mice have been observed to generate various cells in the small intestine, such as enterocytes, goblet cells, Paneth cells [25], and even atypical enteroendocrine cells [26], and successfully restored intestinal nerve function and blood vessels [27, 28]. Human pluripotent stem cells were directed to differentiate into 3D‐like tubules in a microfluidic device, which not only produced markers of these cells, but over the course of 2 weeks developed barrier function and apical‐to‐base polarity, and also expressed adult gut markers such as the efflux transporter P‐glycoprotein and the drug metabolism enzyme cytochrome P4503A4 [29]. Functionally showing similar functional features to the mature gut, it is maintained for a long time, has mature epithelial and mesenchymal layers, and exhibits epithelial functions for peptide absorption, fluid secretion, and intestinal‐like peristalsis to histamine and atropine, similar to the mature human gut [30, 31]. Human colon organoids(HCOs) transplanted into the renal capsule of mice developed at 6–10 weeks resembled a normal colon morphology and expressed colon‐specific markers (SATB2) and hormones (insl5). In addition, hCOs were enriched in the GLP‐1 and PYY‐expressing cells that are the most abundant in the colon. It further matured into an adult colon after transplantation [32]. In vivo implantable human intestinal organoids (HIOs) generated in vitro from human embryonic stem cells (escs) or induced pluripotent stem cells (ipscs) implanted in mice can grow in mice to form various structures of the gut and exhibit digestive function [33]. Intestinal organoids derived from resident adult tissues can arise from anywhere in the gut: including the stomach, duodenum, jejunum, ileum and colon. A recent study showed that intestinal endocrine cells generated from the duodenum, jejunum and ileum of Neurogain3(Ngn3)‐EGFP mice exhibited an in vivo expression pattern and secreted related hormones, such as growth hormone‐releasing peptide, cholecystokinin, secretin and glucagon, similar to those in native tissues [34]. These cells also maintain self‐renewal and cell diversity in long‐term culture [35]. Because the advantages of organoids in simulating intestinal physiology, intestinal organoids and their extension have been widely used in the study of intestinal physiology and diseases, especially in the study of the intestinal flora. The concept of “Organoids on a chip” opens up the possibility to study host–microbe interactions and long‐term infection, by infecting mini‐intestinal tubes with Vibrio parvus. Live‐cell microscopy revealed that open organoids support Vibrio parvum to complete the life cycle and long‐term growth without compromising tissue integrity. We observed continuous production of newly formed oocysts for at least 4 weeks. Based on traditional organoids, this device created a scaffold that is permeable to gases, nutrients and macromolecules, which facilitates the effective adhesion, proliferation and differentiation of intestinal stem cells (ISCs) and restricts the growth of ISCs to a predefined shape. This scaffold not only produced typical, rare and special cells but also retained the key physiological characteristics of the intestine. The scaffold also had significant regenerative capacity [36] and better simulated the oxygen gradient required by the intestinal flora with prolonged co‐culture time, and accurately represented the types of crypt cells [37]. Similarly, VIVIanSwli has made great achievements in understanding gastrointestinal inflammation and infectious diseases with “mini‐guts” [38]. Recently, the gut microbiota has been shown to play a major role in intestinal homeostasis and is associated with many intestinal diseases, such as inflammatory bowel disease [39, 40], colorectal cancer [41, 42] functional dyspepsia and other diseases [43]. However, due to the complexity of the gut, developing a suitable in vitro model to reproduce the mechanism of microbe–host interactions in vivo is difficult. Based on the above‐mentioned advantages of organoids, it seems necessary to incorporate biota into gastrointestinal organoids.

### 3.2 A Model of Intestinal Organoids

**FIGURE 2:**
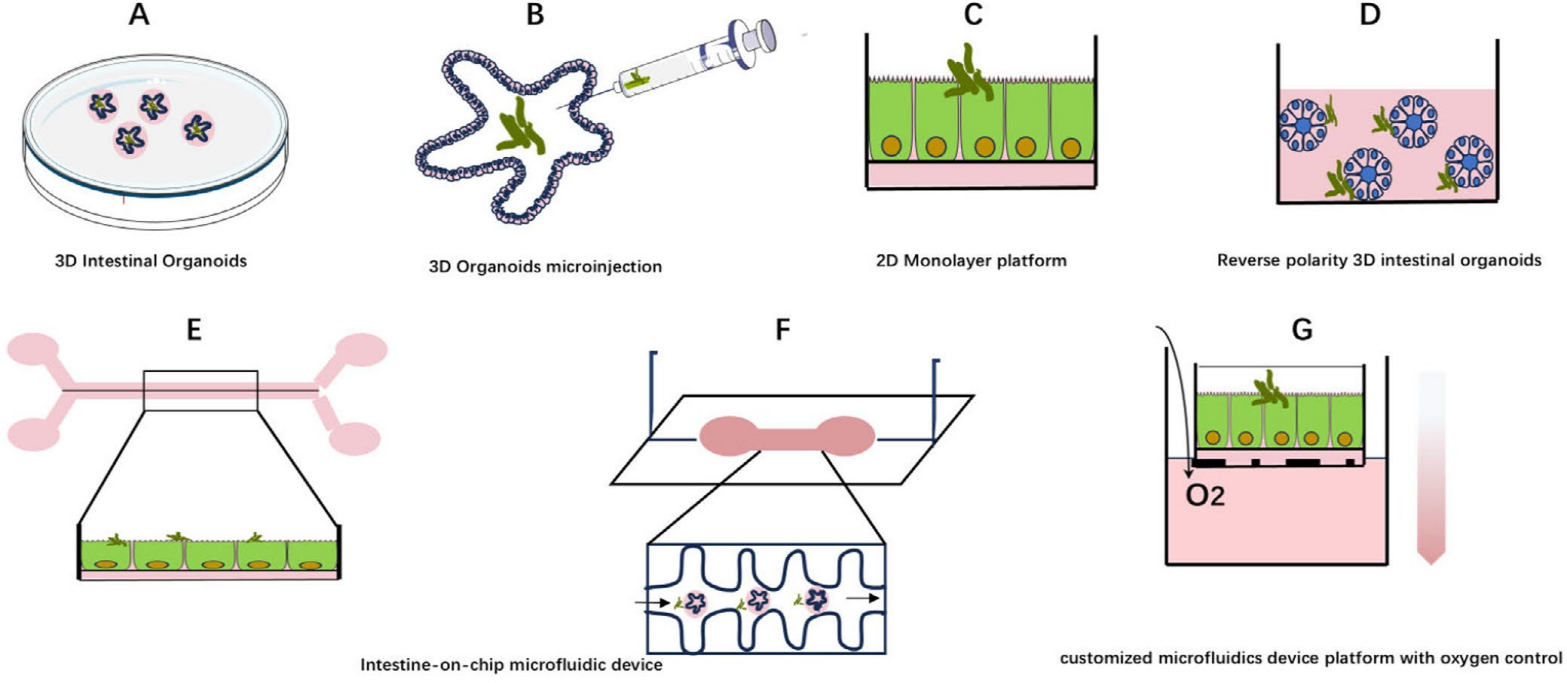
Co‐culture model of intestinal organoids and microorganisms. (A) Conventional 3D intestinal organoids. (B) Microinjection of 3D organoids with bacteria. (C) 2D Monolayer platform allows high throughout paired measurements of paracellular and transcellular permeability and the development of organotypic co‐culture by the addition bacteria. (D) The apical out 3D organoids allow the exposure of biotics to the apical surface by adding bacteria directly to culture media. (E–G) These individual elements can be incorporated into single intestine‐on‐chip models to increase their complexity and provide a more faithful reconstruction of the intestinal tissue and microenvironment.

Organoids are valuable models that can be used to study the gut microbiota (Figure 2; Table 1).

**TABLE 1:** Different intestinal organoid model.

Types	Matrix	Disease
(1) 3D organoids (2) 3D organoids microinjection (3) Reverse polarity 3D intestinal organoids	Extracellular support matrix and medium supplemented with pro‐intestinal growth factors. Wnt‐3a (W), epidermal growth factor EGF (E), Noggin (N) and Rspondin 1 (R) (WENR vector) and regulating stem cell niche signalling pathways, including Wnt, bone morphogenetic protein (BMP) and Notch and other desired vectors [44])	Enterohemorrhagic *Escherichia coli* (Allaire et al. 2018), a study of multiple microorganisms infecting the epithelium [45]
2D Monolayer platform	2D Monolayer platform: Ecm‐coated porous membranes or plates, when grown in the presence of intestinal growth factors, further expansion of organoid monolayer into polarised monolayer, thin layer of type I collagen gel supplemented with subcutaneous myofibroblasts (ISEMF), and formation of more differentiated monolayer or more self‐regenerative monolayer are controlled by the addition of specific growth factors to the medium [46, 47]	Human cholera infection, Listeria and A71 virus studies [48, 49]
(1) Intestine‐on‐chip microfluidic device (2) customised microfluidics device platform with oxygen control	Engineering derived technology: (1) Microfluid‐based engineering scaffolds: Microfluidic device composed of PDMS (microfabricated polydimethylsiloxane) column (Whitesides 2006), (2) 3D biomimetic system based on microfluidic technology [50]	Anaerobic infections [51], *Enterococcus faecalis* [52]

Compared with cell lines, organoids are more suitable for studying host microbial interactions because organoids are more similar to the cells and environment present in the body. Initially, intestinal organoids are 3D‐like closed structures, so it is difficult to use them to study the intestinal microbiota. Linking organoids to 2D molecules or microinjecting individual patients are new ideas. Organoid‐based 2D monolayers were utilised to recapitulate the typical features of human cholera infection [48]. At the same time, human 2D primary organoid‐derived epithelial monolayer models were successfully used for Listeria and viral A71 studies [49]. In addition to studies in humans, porcine intestinal‐like substances have been used to study diarrhoea caused by the interaction of toxin‐producing *Escherichia coli* (ETEC) with the epithelium, with similar results as that obtained in vivo, where enteroid‐based 2D monolayer molecules show adhesion of ETEC [53]. Equine enteroid‐derived 2D monolayer molecules derived from equine enteroid showed a stimulatory response to microbial mimics, producing similar proinflammatory and anti‐inflammatory responses [54]. However, this destroys the 3D structure of the organoids, thereby reducing the physiological relevance. Microinjection methods can be used to successfully inject single or several microorganisms into organoids to study host–microbe interactions [55], but this method requires high requirements for both technology and researchers, is difficult to implement, and is an “invasive operation”. However, the 3D organoid model of reverse polarity not only maintains the original spatial structure and differentiates a variety of enterocyte types but also makes apical epithelial cells easily accessible, and microbial–host interactions can be studied by simply adding microorganisms to culture medium [56]. This model has been used to explore the preferred infection sites of *Salmonella typhimurium* and *Listeria monocytogenes* , and triple culture of organoids, *Lactobacillus casei* , and *Bifidobacterium longum* can promote organoid barrier formation and increased mucin production, and can be used to study epithelial infection by a variety of microorganisms [45]. However, due to apical exposure, it does not seem to be the best culture condition for anaerobes. Lana. Williamson's team developed a semiautomated, high‐throughput microinjection system to achieve high‐throughput sampling of bacteria and accurate injection of bacteria into organoid lumen. Functional studies of microbial communities and anaerobes derived from transplanted faeces have been performed [57]. In addition, 3D porous protein scaffolds compartmentalise intestinal tissues to form a mucus layer, which is essential for the growth of intestinal microbiota. Moreover, they mimic the oxygen gradient in vivo, exhibit a depth‐wise hierarchical oxygen distribution in the luminal direction, gradually decreasing to highly hypoxic conditions, and can be cultured for a long time in vitro. These findings support the in vitro culture of the gut microbiota and the potential to reproduce the in vivo effects of anaerobes dominant gut microbiota [58]. Furthermore, a 3D scaffold system produced by culturing of the intestinal epithelium with a complex living symbiotic gut microbiome, including obligate anaerobes, was developed to establish two physiologically relevant oxygen gradients through “up” and “flip” orientations [59]. The majority of gut bacteria are anaerobic; thus, this model overcomes the limitations of traditional aerobic models. However, the human gut is unblocked, and transport in and out of the gut is essential for gut physiology. An intestinal organoid fluidics chip is a three‐layer microfluidic device that creates an intraluminal and extraluminal long‐term continuous luminal flow that can be used to introduce intestinal microbes, monitor microbial dynamics in real time, and detect the presence of isolated microbes, dissolved waste and metabolites in luminal effluent [60, 61]. Ginga et al. also developed perfusion systems for real‐time imaging analysis of the luminal contents of human intestinal organoids and microbial populations, which could be used to simulate bacterial infection [51]. At the same time, microfluidic channel human colon chips can generate a mucus bilayer in vitro, which may be able to expand significantly without losing barrier function or structural stability and replicate the dynamic changes in mucus layer thickness caused by the inflammatory mediator PGE2 in vivo, which can also be quantified and analysed [62]. Species‐specific microbiota and individual bacteria can be cultured in a colon microarray to directly quantify the specific effects of bacteria on epithelial cell adhesion, tight junctions, barrier function, mucus production and cytokine release. Colon microarray showed different responses to pathogenic *Salmonella typhimurium* with or without colonisation by the normal complex microbiota. The potential for a single type of commensal bacteria to produce beneficial and harmful properties has been highlighted, for example *Enterococcus faecalis* , a bacterium that promotes host tolerance to infection, also induces epithelial detachment in small areas [52]. Claudia Beaurivage's group also attempted to incorporate other physiological conditions into the model, for example they incorporated macrophages into the model and successfully used it to the study inflammatory bowel disease [63].

### 3.3 Diseases

**FIGURE 3:**
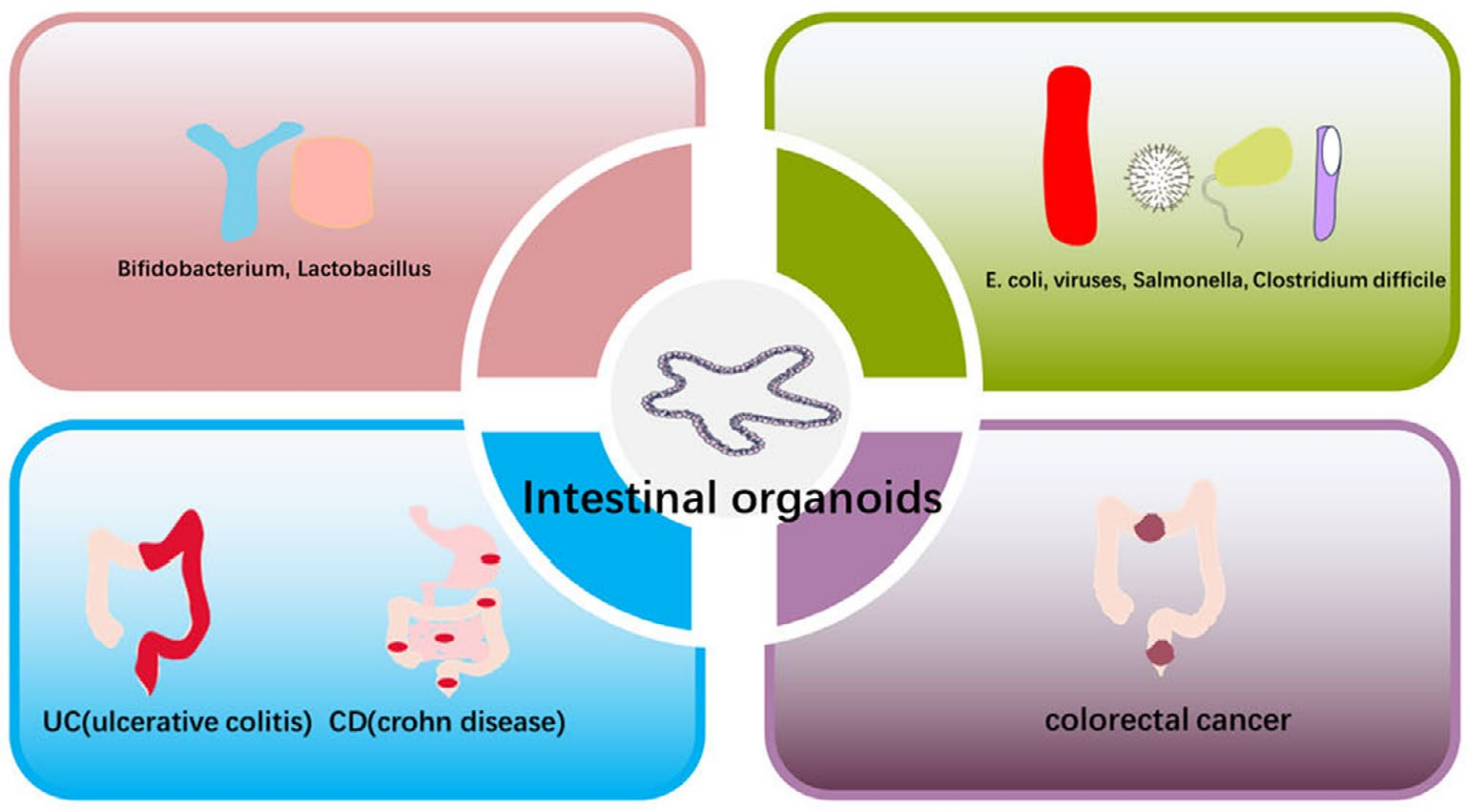
Intestinal organoids and intestinal diseases. Intestinal organoids can be used to study various intestinal diseases, such as inflammatory bowel disease and colorectal cancer.

The gut microbiota has specific functions in host nutrient metabolism, exogenous substance and drug metabolism, maintenance of the structural integrity of the intestinal mucosal barrier, immune regulation and resistance to pathogens, and is affected by age, diet, use of antibiotics and probiotics, and has different diversity [64]. Microbes are integral to the gut and help maintain its normal function. As mentioned above, organoids are the most valuable tools to model microbe–host interactions. Some researchers have studied the effect of co‐culture with gut organoids and commensal bacteria such as Lactobacillus [65]. N‐carbamoylglutamate (NCG), a metagenic metabolite of Lactobacillus reutella strain DS0384, was found to promote the maturation of the human intestinal organoid epithelium, stimulate the proliferation of intestinal stem cells during intestinal epithelial regeneration and protect human intestinal organoids from IFNγ/ TNFα‐induced inflammation in organoids. This finding is consistent with in vivo experiments in mice [66]. It also plays an important role in promoting the growth and development of intestinal stem cells and protecting the intestine, such as the mucin degrading agent *Akkermansia muciniphila* , and is involved in the expression of genes in intestinal epithelial cells. For example, *Akkermansia muciniphila* and *Faecalibacterium prausnitzii* are common and abundant commensal intestinal bacteria in the human gut. In studies on the co‐culture of *Akkermansia muciniphila* , *Faecalibacterium prausnitzii* bacteria and their metabolites, such as short‐chain fatty acids (SCFAs), butyrate, acetate and propionate, with organoids, *Akkermansia muciniphila* and propionate played more important roles in the regulation of various transcription factors and genes involved in cellular lipid metabolism and growth than *Faecalibacterium prausnitzii* , which may indicate that intestinal bacteria have differential regulation of intestinal epithelial cell function [67]. The microbiome can also help resist pathogen infection by inducing host tolerance furthermore the commensal strain, *Enterococcus faecium* , protects the colonic epithelium from *Salmonella typhimurium* induced damage, while also promoting the integrity of tight junctions. In addition to commensal bacteria, other pathogenic bacteria and viruses have also been studied. Such as pathogenic bacteria *Escherichia coli* , Salmonella, *Clostridium difficile* , Cryptosporidium and viruses, to understand the effects of bacterial pathogens and viruses on intestinal epithelial cells. Colonisation of human colonic organoid monolayers by enterohemorrhagic *Escherichia coli* (EHEC) reduces the amount of intestinal mucus, disrupts microvilli structure and can lead to epithelial damage, as well as the entry and infection of bacteria in epithelial cells [68]. This mechanism was found to involve reduction of brush border resident procadherin 24 (PCDH24) by the EHEC protease EspP. It was demonstrated that the model system was able to reveal the molecular mechanisms responsible for early intestinal colonisation, and that the presence of human microbiome metabolites such as 4‐methylbenzoic acid, 3, 4‐dimethylbenzoic acid, caproic acid and heptanoic acid induced greater epithelial damage, flagellin was a key regulator in this process [52, 69]. These metabolites mainly induced β‐defensin 2 (hBD2) expression and decreased IL‐8 levels [70]. The addition of *Salmonella typhimurium* to organoid culture media results in epithelial detachment, decreased staining of tight junctions and increased release of chemokines (CXCL1, CXCL2 and CCL20) [52], furthermore bacteria‐ infected organoids display pathophysiological features that are not available in other in vitro models. By establishing an in vitro human model of *Clostridium difficile* infection or *Cryptosporidium parvum* infection, direct contact between the intestinal organoid lumen and bacteria was achieved using microinjection techniques [71, 72]. Difficult clostridium persist in such organs and produce the TcdA and TcdB, lower on DRA (downregulated in adenoma, Cl−/[Formula: see text] exchanger) [73] and inhibition of the Na(+)/H(+) exchanger 3 (NHE3) [71] altering the gut environment and the composition of the microbiome. Cryptosporidium can propagate and complete its life cycle in human intestinal and lung organoids [74], which is possible only in suitable hosts, competition of this cycle requires conditions such as an “air–liquid interface” (ALI), where cultivation and genetic processing can be accomplished [75]. Enterovirus is also an important pathogenic factor. It is difficult to culture and study them in vitro before, so developing a culturable in vitro model is the key. Ettayebi K et al. successfully grew norovirus in monolayer cultures of human enterograsi [76] and supported its efficient replication [77]. The same can also support the culture of rotavirus [78] and SARS‐CoV‐2 [79]. Organoids have been used to construct personalised infection models of viruses as a way to study viral virotherapy (Figure 3).

#### 3.3.1 Human‐Restricted Pathogens

The induction of human intestinal organoids is a new experimental model for studying the infection of human‐limiting pathogens such as Shiga toxin‐producing *Escherichia coli* (STEC) in the intestine. Shiga toxin can cause severe complications in humans, such as hemolytic uremic syndrome (HUS). It was found that commensal *E. coli* could grow in large amounts in the intestine without causing damage, while *E. coli* infection could cause rapid loss of the epithelium, activate the innate defence and immune responses, and increase the number of neutrophils, which were related to the occurrence and death of HUS [80]. Moreover, intestinal organoids can mimic the complex intestinal tissue response to Shiga toxin injury. We observed that STX2a not only caused changes in epithelial structure but also protein synthesis. Apoptosis was also induced in both epithelial and mesenchymal cells. Epithelial‐mesenchymal transition (EMT), a marker of E‐mucin, was also observed [81]. *Salmonella typhi* invades intestinal cells by rearranging the enterocyte cytoskeleton, reproduces by producing VI antigen to evade the host immune response, and forms vesicles that dot not activate the host's adaptive immune system to cause effective infection [82]; however, Lactobacillus can reduce the damage to intestinal organoids caused by Salmonella. In organoids, the epithelial differentiation is affected by inhibiting the overexpansion of goblet cells and Panes cells, through contact with Toll‐like receptor 2 (TLR2), which ameliorates overactivation of the Wnt/white ta‐catenin pathway by Salmonella [83]. The same α‐defensins protect intestinal epithelial cells by killing *Salmonella typhimurium* . Salmonella causes a wide variety of intestinal diseases.

#### 3.3.2 Inflammatory Bowel Disease (IBD)

IBD is a type of intestinal immune disorder caused by environmental factors that act on genetically susceptible people under the action of intestinal microorganisms; this disorder damages the intestinal mucosal barrier and leads to persistent intestinal mucosal inflammation, including Crohn's disease and ulcerative colitis. Intestinal flora imbalance can cause an abnormal intestinal immune response, and faecal microbiota transplantation is an effective way to treat this disease [84]. However, the immune mechanism of intestinal microorganisms is poorly understood, which makes it difficult to cure this disease. The discovery of intestinal organoids from the lack of appropriate research models has undoubtedly brought infinite hope to the research in this field. Several kinds of organs and organoid‐derived models have been developed to study the immune mechanism of intestinal microorganisms in inflammatory bowel disease. Organoids are closer to cellular physiology in vivo, and the interaction between microbes, epithelial cells and monocytes has been a research hotspot since the development of organoids. Based on this, Ibrahim M Sayed et al. identified ELMO1 (phagocytosis and cell motility protein 1) as an important proinflammatory factor in patients with IBD, serving as a microbial sensor in epithelium and phagocytes that triggers inflammatory signals and causing elevation of proinflammatory factors, such as MCP‐1, providing a biomarker for early detection of inflammation [85]. The co‐culture of bioengineered 3D sponges with colonic organoids and macrophages can reflect the epithelial–immune interactions in IBD. *E. coli* induces inflammation, reduces the epithelial coverage, promotes the migration of macrophages and increases the expression of inflammatory factors, all of which can be observed in this system, these findings provide another idea for the treatment of inflammatory bowel disease [86]. A hybrid trigger consisting of bacterial LPS and cytokine IFN‐γ was applied to the microfluidic model OrganoPlate platform, which revealed the general inflammatory state of patients with inflammatory bowel disease and exhibited macrophage function. The model allowed intestinal epithelial cells to be exposed to TPCA‐1 in a polarised manner. However, TPCA‐1 prevents the phosphorylation of NF‐κB (IκB) inhibitor, which is not only detected in the intestinal mucosa of patients with active inflammatory bowel disease but also alleviates clinical symptoms [63]. The maintenance of normal intestinal epithelial cell function is extremely important for maintaining intestinal ecological balance in the intestine. Moreover, intestinal organoids can be used as models to screen drugs for inflammatory bowel disease. Infliximab (a drug for clinical treatment of inflammatory bowel disease) can attenuate the damage of intestinal mucosa caused by TNF‐α and TGF‐β, and SB431542, an inhibitor of TGF‐β, can inhibit this effect [87].

#### 3.3.3 Colorectal Cancer

Colorectal cancer (CRC) is the third‐most common malignancy and the second‐leading cause of cancer death in the world. There are many risk factors associated with CRC carcinogenesis, including genetic alterations, lifestyle and environmental factors. Over the past decade, the gut microbiota has been shown to play a key role in CRC development. The use of bacteria as cancer therapy dates back a long way, and GillianM. Mackie et al. also found that attenuated STm therapy was effective in a mouse model of primary bowel cancer [82]. However, the direct role of bacteria in the occurrence of oncogenic mutations has not been determined, and the establishment of high‐quality in vitro research models is crucial. The mutation characteristics of PKS *E. coli* toxin‐promoting CRC development have been found by co‐culture of PKS *E. coli* with intestinal organoids. SBS‐PKS (base substitution) and ID‐pks (insertion or deletion) are the result of mutations, and these mutations are present in the CRC genome [88, 89], which are critical for early prevention of CRC, such as targeted depletion of genotoxic bacteria, interference with mutagenesis in *Escherichia coli* and production of PKS bacteria. Some probiotics such as *Streptococcus thermophilus* and *Lactobacillus rhamnosus* have shown anti‐cancer properties. *L. gallinarum* inhibits the development of human CRC‐derived organoid tumours [90]. All these are important approaches to the treatment of cancer. Similarly, intestinal organoids, especially mouse‐ and patient‐derived organoids, are the best in vitro models for colorectal cancer drug screening [91].

## 4 Therapeutic Aspects of Intestinal Organoids

In addition, the treatment of intestinal organoid transplantation is also booming. Intestinal organoid transplantation not only plays a therapeutic role in reducing intestinal ischemia–reperfusion injury but also can be used for the treatment of short bowel syndrome [13]. For example, researchers transplanted intestinal organoids into mice with intestinal ischemia–reperfusion and found that organoids could mediate M2 macrophage polarisation and enhance mucosal recovery by secreting L‐malic acid (MA), possibly by inducing M2 polarisation through MA in a SOCS2‐dependent manner [92]. Especially, it is of great significance in the treatment of inflammatory bowel disease. For example, intestinal organoids containing poly (lactic‐co‐glycolic acid) nanoparticles can be used to treat inflammatory bowel disease, and intestinal organoids can be used as drug‐loaded carriers to deliver drugs to the inflamed areas to achieve therapeutic purposes [93]. The protective sulfamic is reduced in the inflammatory area of UC, and the transplantation of mouse cecal organoids (with increased expression of sulfamic in the cecal area of mice) into the injured epithelium of the distal colon found that the expression of sulfamic can be increased after transplantation to achieve a protective effect [94]. Not only that, the use of patient‐derived intestinal organoids can also be used to evaluate the ability of epithelial regeneration in Crohn's disease [95], and intestinal organoid transplantation therapy may constitute a new therapeutic strategy to rebuild mucosal barrier function in critically ill patients.

## 5 Retrospect and Prospect

In terms of simulating human physiology, organoids have various advantages. So far, a variety of human organoids have been invented, such as heart, liver, kidney, intestine, etc., and we mainly focus on intestinal organoids. In the past few decades, studies of the GI tract have been mainly based on monolayer cell cultures and mouse models, which have many shortcomings in simulating human GI physiology. The generation of intestinal organoids has brought infinite possibilities for its research, summarising many aspects of intestinal physiology. In terms of structure, it contains a variety of intestinal epithelial cells, and in terms of function, it has intestinal absorption, secretion and other functions. Taking the study of the gut to the next level, particularly in terms of gut microbiota and intestinal diseases, provides new ways to study host–microbe interactions. Combining patient‐specific models with the use of organoids will enable researchers to advance the development of personalised medicine and find new treatments for infectious diseases, including infection‐related cancers.

## Author Contributions

**Ya Deng:** writing – original draft (equal), writing – review and editing (equal). **Xiaolu Yuan:** writing – original draft (equal), writing – review and editing (equal). **XianMin Lu:** investigation (equal). **Jiangbo Wu:** methodology (equal). **Chen Luo:** investigation (equal). **Ting Zhang:** software (equal). **Qi Liu:** investigation (equal). **Siqi Tang:** investigation (equal). **Zhuo Li:** conceptualization (equal), formal analysis (equal). **Xingyi Mu:** software (equal). **Yanxia Hu:** investigation (equal), methodology (equal), supervision (equal). **Qian Du:** supervision (equal). **Jingyu Xu:** funding acquisition (equal), methodology (equal), resources (equal). **Rui Xie:** funding acquisition (equal), methodology (equal), project administration (equal), resources (equal), supervision (equal).

## Ethics Statement

The authors have nothing to report.

## Consent

We have obtained consents to publish this paper from all the participants of this study.

## Conflicts of Interest

The authors declare no conflicts of interest.

## Data Availability

The authors have nothing to report.
